# Has industry funding biased studies of the protective effects of alcohol on cardiovascular disease? A preliminary investigation of prospective cohort studies

**DOI:** 10.1111/dar.12125

**Published:** 2014-03-07

**Authors:** Jim McCambridge, Greg Hartwell

**Affiliations:** London School of Hygiene & Tropical MedicineLondon, UK

**Keywords:** alcohol, alcohol industry, bias, cardiovascular disease, health benefits

## Abstract

**Introduction And Aims:**

There have been no previous quantitative analyses of the possible effects of industry funding on alcohol and health research. This study examines whether findings of alcohol's protective effects on cardiovascular disease may be biased by industry funding.

**Design and Methods:**

Findings from a recent systematic review of prospective cohort studies were combined with public domain data on alcohol industry funding. The six outcomes evaluated were alcohol's effects on cardiovascular disease mortality, incident coronary heart disease, coronary heart disease mortality, incident stroke, stroke mortality and mortality from all causes.

**Results:**

We find no evidence of possible funding effects for outcomes other than stroke. Whether studies find alcohol to be a risk factor or protective against incident stroke depends on whether or not there is possible industry funding [risk ratio (RR) 1.07 (0.97–1.17) for those without concern about industry funding compared with RR 0.88 (0.81–0.94)]. For stroke mortality, a similar difference is not statistically significant, most likely because there are too few studies.

**Discussion and Conclusions:**

Dedicated high-quality studies of possible alcohol industry funding effects should be undertaken, and these should be broad in scope. They also need to investigate specific areas of concern, such as stroke, in greater depth. *[McCambridge J, Hartwell G. Has industry funding biased studies of the protective effects of alcohol on cardiovascular disease? A preliminary investigation of prospective cohort studies.* Drug Alcohol Rev *2015;34:58–66]*

## Introduction

The apparent protective effects of alcohol on cardiovascular and other diseases make it unlike other drugs, such as tobacco. Concerns about the validity of these findings, for example, in relation to misclassification bias of formerly heavier drinkers who have stopped, have existed for approximately 30 years [1,2]. In addition to the well-known problems implicit in making causal inferences on the basis of observational data, beyond cardiovascular disease many of the claimed health benefits have no plausible biological mechanisms nor obvious relationships to each other [3]. This is all the more curious as reliable exposure measurement of patterns of drinking over time is complex, and may be weak in many of the primary studies [4]. A recurrent finding in meta-analytic studies of alcohol's protective effects on cardiovascular disease is the striking levels of unexplained heterogeneity between studies [5,6].

Disclosure of internal tobacco industry documents—mandated by the courts during litigation in the USA—has revealed a history of subversion of science spanning decades [7]. It has previously been suggested that alcohol industry-funded reviews are ‘those studies which reported more enthusiastically about the potential cardio-protective nature of moderate alcohol use’ [1]. One recent ‘consensus document’ in this area brings together a wide range of Italian medical and academic bodies under the auspices of the Nutrition Foundation of Italy, yet it does not identify the foundation as an industry body in the paper, or contain any declarations of conflicts of interest [8].

Substantial grounds for concern about industry influence on alcohol research [9,10] have not led to quantitative investigations of possible funding effects. We thus sought to examine whether findings from a recent systematic review of prospective cohort studies of alcohol's protective effects on cardiovascular disease may be biased by industry funding.

## Methods

For this preliminary investigation, we examined a recent systematic review of prospective cohort studies [11] and obtained the primary reports of all 84 included studies. We were aware that, in the case of tobacco, industry lawyers had routinely edited scientific papers written by industry-funded scientists, deleting acknowledgements of industry sponsorship [7]. In addition to declarations of funding sources in the 84 reports themselves, we gathered data from industry research funding websites (see Babor [10]), peer-reviewed papers and other public domain data sources, such as university websites, and contacted authors from all papers for additional information [64/80 email replies (80%), two papers no active email addresses found, two papers all authors deceased]. We categorised individual studies as described in Box 1, double coding blindly any study where there was any doubt about categorisation. It will be seen that we assumed the absence of any funding information (i.e. category 5) to indicate no concern. As this is a questionable decision, we undertook a sensitivity analysis to interrogate this assumption. Our approach to possible industry funding was not restricted to individual papers or to individual authors; we took any evidence of industry funding for any purpose at any time by any author (including travel) and university receipt of any alcohol industry funding for health research at any time to indicate some level of concern.

**Box 1. Levels of concern about industry funding of research**

**High level of concern**

1. Declaration in paper of industry funding of study being reported
2. Any evidence of any author in receipt of undeclared industry funding (from publicly available data sources)

**Moderate level of concern**

3. Declaration in paper of any author in receipt of industry funding for any other purpose, such as travel, at any time
4. Any evidence of institutional funding concerns (i.e. industry funding health-related studies by other researchers in the same institution of any author, from publicly available data sources) at any time

**No concern**

5. No funding information in paper, nor replied to email seeking further information
6. No funding information in paper, replied to give assurances ruling out industry funding
7. Declaration in paper of non-industry funding sources, no reply to email seeking further information
8. Declaration in paper of non-industry funding sources, replied to give assurances ruling out industry funding

Ronksley and colleagues [11] compared drinkers with non-drinkers in prospective cohort studies and investigated cardiovascular disease mortality, incident coronary heart disease, coronary heart diseasemortality, incident stroke, stroke mortality and all-cause mortality, with subsets of studies providing data for each outcome. We calculated relative risk estimates (presented as effect size in the figures) and their confidence intervals for the associations with alcohol consumption, pooled in random effects models (using the method of DerSimonian and Laird with the metan command in stata; Statacorp LP, College Station, TX, USA) stratified by level of concern about industry funding, as shown in Box 1. We re-ran the metan command to test for statistical significance of differences between strata. We combined moderate and high levels of concern because there were few instances of the latter for some outcomes and ran the analyses for both the two (some concern vs. no concern) and three (no, moderate and high levels of concern) groups of studies. For presentational reasons, we offer the data for the some versus no concern contrast as there was generally little difference in outcomes between the studies with high levels of concern about industry funding and those categorised as moderate concern. For the sensitivity analysis, we investigated the effect of the absence of information (category ‘5’) being taken as constituting some level of concern rather than no concern. This left the ‘no concern’ category to include only those studies where some specific evidence existed for a lack of industry funding (e.g. assurances received from the author ruling out industry funding or a declaration in the paper itself of non-industry-funding sources).

## Results

**Figure 1:**
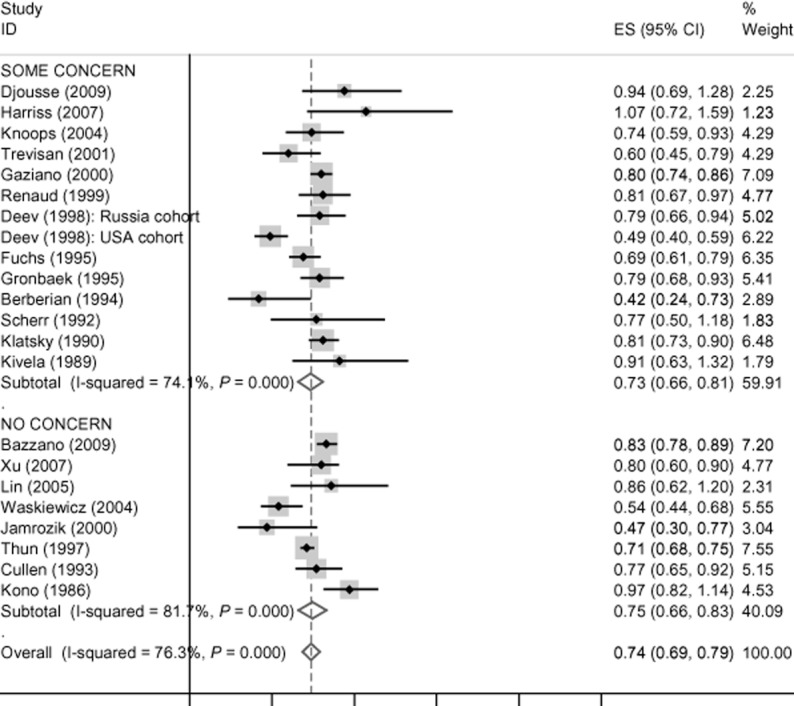
Cardiovascular disease (CVD) mortality findings by concern about industry funding. CI, confidence interval.

**Figure 2:**
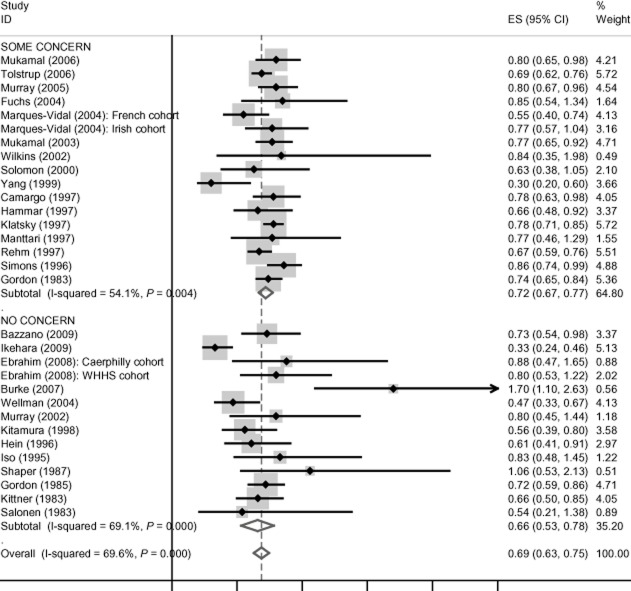
Coronary heart disease (CHD) incidence findings by concern about industry funding. CI, confidence interval.

**Figure 3:**
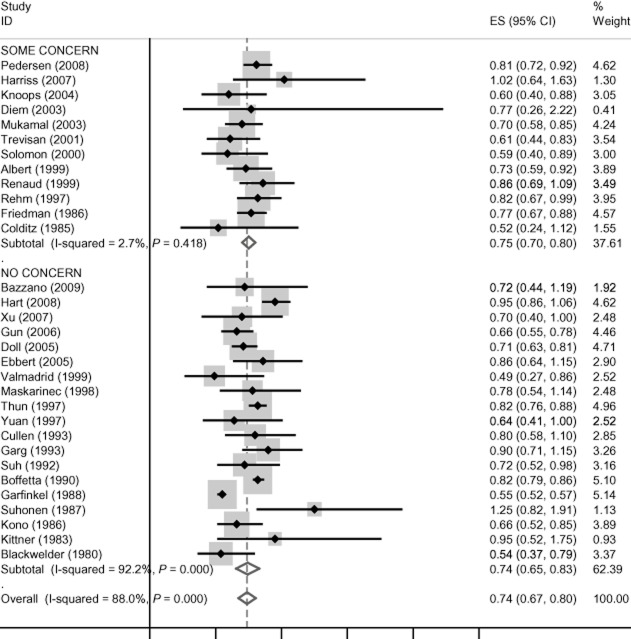
Coronary heart disease (CHD) mortality findings by concern about industry funding. CI, confidence interval.

**Figure 4:**
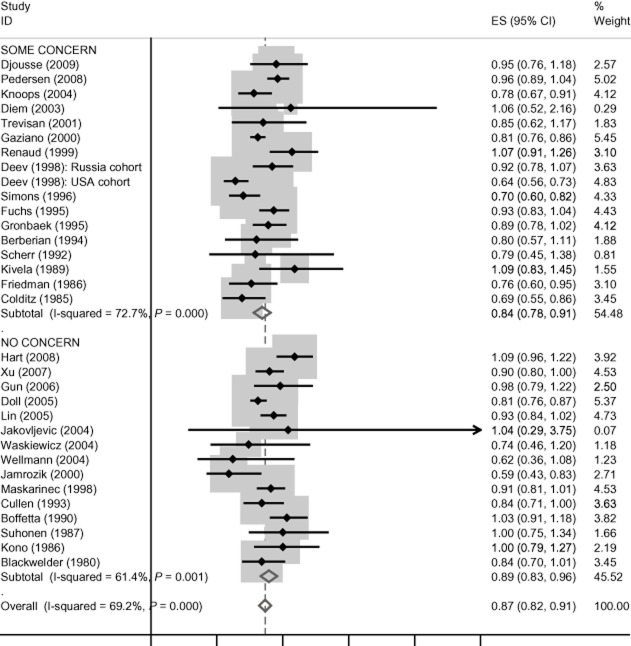
All-cause mortality findings by concern about industry funding. CI, confidence interval.

**Figure 5:**
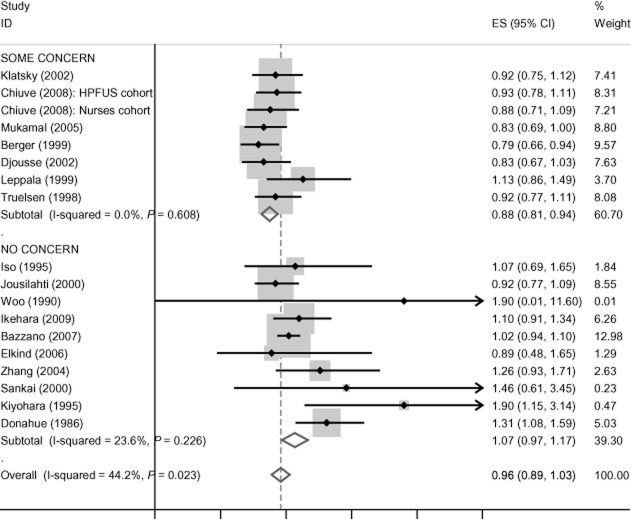
Stroke incidence findings by concern about industry funding. CI, confidence interval; HPFUS, Health Professionals Follow-Up Study.

There is no evidence of funding effects for cardiovascular disease mortality, incident coronary heart disease, coronary heart disease mortality and all-cause mortality, and these data are presented in Figures 1–4. Concern about industry funding is, however, associated with observed outcomes for stroke. Alcohol consumption is found to be *protective* against stroke incidence in the group of eight studies where there is concern about industry funding, whereas it is found to be a *risk factor* in the group of 10 studies where concern about industry funding is absent, with little heterogeneity within the two groups; see Figure 5. Differences between these pooled effects are statistically significant [χ^2^ = 9.75 (1 d.f.) *P* = 0.002].

When splitting the high and moderate levels of concern for the stroke incidence findings, we found that the pooled effects for the two studies with high levels of concern about industry funding were somewhat less discrepant from the studies with no concern compared with those with moderate levels of concern [risk ratio (RR) 0.92 (0.79–1.05) compared with RR 0.86 (0.79–0.93)]. For the sensitivity analysis, whereby category ‘5’ was taken as constituting some level of concern rather than no concern, no differences were observed in the point estimates presented in Figure 5. This applied both to the analyses comparing some concern with no concern and to those when the groups were split into moderate and high levels of concern, with only minor changes to confidence intervals in each instance.

**Figure 6:**
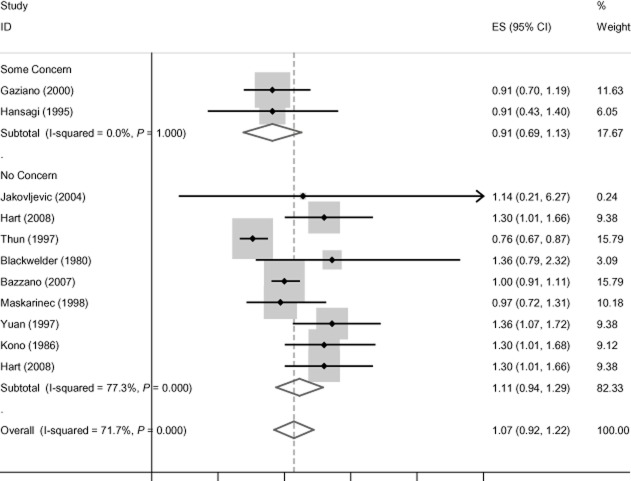
Stroke mortality findings by concern about industry funding. CI, confidence interval.

Although there are few studies of stroke mortality and only two with funding concerns, a similar pattern is apparent in the pooled relative risk estimates for that outcome (see Figure 6). The difference is not statistically significant [χ^2^ = 1.94 (1 d.f.), *P* = 0.163].

## Discussion

This study gives no specific grounds for concern that alcohol industry funding has biased what is known about the protective effects of alcohol on cardiovascular disease, apart from with regard to stroke. Our investigation provides evidence that findings from studies evaluating associations between alcohol consumption and incident stroke vary considerably according to whether or not there is concern about industry funding. This difference is unlikely to have arisen by chance, even allowing for multiplicity of analyses and even though similar evidence was absent for other cardioprotective outcomes. This set of findings provides grounds for a more generalised concern that there is a need to evaluate further whether observed heterogeneity among studies of the health benefits of alcohol may be in part due to industry funding. The data patterns described here are curious as it is not obvious *prima facie* why findings for stroke might be discrepant from other possible protective effects on cardiovascular disease outcomes.

This study has clear limitations. It is a brief review of published findings in this literature as a whole, and no attempt has been made at this stage to investigate the detailed content of individual primary studies. The validity of the findings is contingent upon correct classification of funding concern, and our measure is neither validated nor capable of detecting well-hidden funding. It incorporates relatively trivial associations with industry, for example, one author only having accepted travel support at some time previously, or one institution having received alcohol industry funding for health research long ago. Note that covert funding will also have led to some misclassification bias, systematically biasing observed associations towards the null. We have not addressed the year of publication, and standards for declarations of conflicts of interest have clearly evolved over time [12]. It was not possible to investigate directly whether previously observed differences in the alcohol risk functions for haemorrhagic stroke (i.e. dose response) and ischemic stroke (j-shaped) for both morbidity and mortality [13] may be partly because of funding effects. Studies where there is concern about industry funding tend to have larger sample sizes than those which do not, and there is evidence that larger studies tend to be less biased [14]. There is also some suggestion of publication bias among those studies included here for which there is no concern about industry funding, as no small studies show strongly protective effects.

The lack of prior quantitative study of the alcohol industry's possible involvement in subversion of science is somewhat surprising [15], given the existence of funding effects is well established, particularly in relation to pharmaceutical companies [16]. Studies indicate that such funding effects are carefully and subtly manufactured by pharmaceutical corporations, selecting certain high-quality primary studies for publication in leading journals and not disclosing others [17]. Funding effects have also been found to bias conclusions in favour of industry in the field of nutrition [18]. There are numerous decisions made in the conduct of any study with potential to bias findings in one direction or another, and such practices are much more difficult to detect than ‘shoddy studies done by mercenary researchers who manipulated methods and data’ [16] as was once believed to be the nature of the influence of corporate funding on health research. Despite sustained efforts in recent years, there has been a limited progress made in the reporting of conflicts of interest in drug trials [19], and progress on observational epidemiological studies should also be expected to be slow, unless actions are taken to accelerate this process.

The alcohol industry is known to have made extensive efforts to subvert the use of science by policy makers 9,10,20–23. We do not know whether the alcohol industry has behaved like the tobacco industry in perpetrating a decades-long conspiracy to subvert the peer-reviewed science base itself [24], though it is important to consider such possibilities. The similarities in the strategic problems faced by both industries are striking [25]: they produce legally available and widely used drugs that will damage the health of, and ultimately kill, significant proportions of their users. It would be surprising if the alcohol industry had ignored the subversion of science as a vehicle for advancing corporate interests, particularly in light of evidence of cross ownership of the tobacco and alcohol industries [26], leading to direct tobacco industry influence on the formation of globally significant alcohol industry strategies [20]. Furthermore, the two industries have previously been found to have worked closely together to thwart the introduction of evidence-based policy measures [27].

This study is designated as preliminary because we suggest it is important to undertake further more sophisticated investigations of the issues covered. Dedicated studies of alcohol industry funding effects can build upon previous work on corporate corruption of science. Investigations of the detailed content of primary studies to identify which, if any, study decisions may be responsible for biasing the health benefits of alcohol should be useful. Although challenging because of the nature of the measurement target, it will be necessary to develop finer-grained reliable measures of industry funding for use in quantitative studies. These could, for example, separately assess the various components we have combined here, such as individual travel and institutional acceptance of funding. Measures which rely upon self-disclosure should clearly note this as a limitation, despite the stringent and sophisticated efforts of journals to enhance transparency by addressing the complexities of conflicts of interest [12]. Studies of funding effects should be broad in scope, investigating where the alcohol industry spends its money on research and where it does not. We are unaware of any significant attention given in the peer-reviewed evidence base to internal alcohol industry research programs [10]. This should be rectified. Experience with the tobacco industry indicates that areas of uncertainty with potential to alter perceptions held by policy makers, as was the case with passive smoking [16], may be of particular interest. For the outcomes investigated here, other than stroke incidence and mortality, alcohol's health benefits appear less likely to have been compromised by funding effects. This study is not designed to assess the validity of these findings, but it may be that funding effects are more likely to exist for stroke because the effects of alcohol on stroke outcomes are more uncertain.
